# An Atypical Presentation of Motor Aphasia: A Case Report and Review of Literature

**DOI:** 10.7759/cureus.19495

**Published:** 2021-11-12

**Authors:** Ananth G, Gaurav Venkat Cuddapah, Amit Shukla, Ramesh Shighakolli

**Affiliations:** 1 Department of Neurosurgery, Kamineni Academy of Medical Sciences and Research Centre, Hyderabad, IND

**Keywords:** broca's aphasia, inferior temporal gyrus, craniotomy, middle temporal gyrus, motor aphasia

## Abstract

Broca's aphasia results due to lesions involving the anterior perisylvian speech area. Patients have intact comprehension and writing but have labored, nonfluent speech with decreased linguistic output. We hereby present a case of a 47-year-old female who was operated on for left ventricular trigonal meningioma by a modified middle temporal gyrus approach and developed motor aphasia as a complication. She had intact comprehension and writing but had decreased linguistic, labored output. It could not be labeled as subcortical aphasia as she had no repetition. Eventually, her aphasia improved completely. Our case is the first of its kind and hence we propose that the posterior middle temporal gyrus area has speech output function, the lesion of which could cause motor aphasia.

## Introduction

Broca's aphasia is a common language disorder, in which people ordinarily have impeded speech and preserved comprehension. In 1861, Broca acknowledged a patient, Louis Victor Leborgne who had a loss of speech and paralysis yet no loss of understanding [1]. Leborgne was withdrawn and merely spoke one word “tan”. After his death, Broca performed an autopsy and found a lesion in the left frontal lobe. Thereafter, he performed autopsies in 12 patients and concluded that the posterior part of the inferior frontal gyrus is a domain of speech, and an insult to it leads to Broca's aphasia. A significant number of Broca's counterparts couldn't agree with his teachings. However, the strongest counterargument was presented by Broca's intern, Pierre Marie who stated that left frontal convolution assumes no job in speech production [2]. Marie proposed that Broca's aphasia was a blend caused by infliction to Wernicke's region and subcortical structures (putamen and pallidum). Dejerine, neurologist and adversary of Marie, defended Broca's unique suggestion contending that harm to Broca's territory was essential for Broca's aphasia, although he likewise included the vital commitment of other structures, like the insula, parietal region, and underlying white matter [3,4]. However, Marie held that Dejerine and others failed to prove a consistent relationship between harm to third frontal convolution and Broca's aphasia [5,6]. The discussion continues to present times, Dronkers et al. utilized high-resolution MRI to examine Leborgne's brain and discovered expansion far beyond the inferior frontal gyrus and included structures such as the insula and inferior parietal lobe [7,8] and disagreements continue about the regions causing Broca's aphasia.

The current consensus is that the harm most likely incorporates parts of Broca's zone and some adjoining structures [7,9,10]. The correct neighboring structures are anyways obscure. In our case, there was an intraventricular mass in the left lateral ventricle which was operated by the middle temporal gyrus approach and totally excised. With this approach, the ordinarily expected aphasias are sensory or transcortical aphasias (commonly sensory), and not motor aphasia. But our patient manifested pure motor aphasia and hence we are proffering an atypical case of developing transient Broca's aphasia as a complication.

## Case presentation

**Figure 1:**
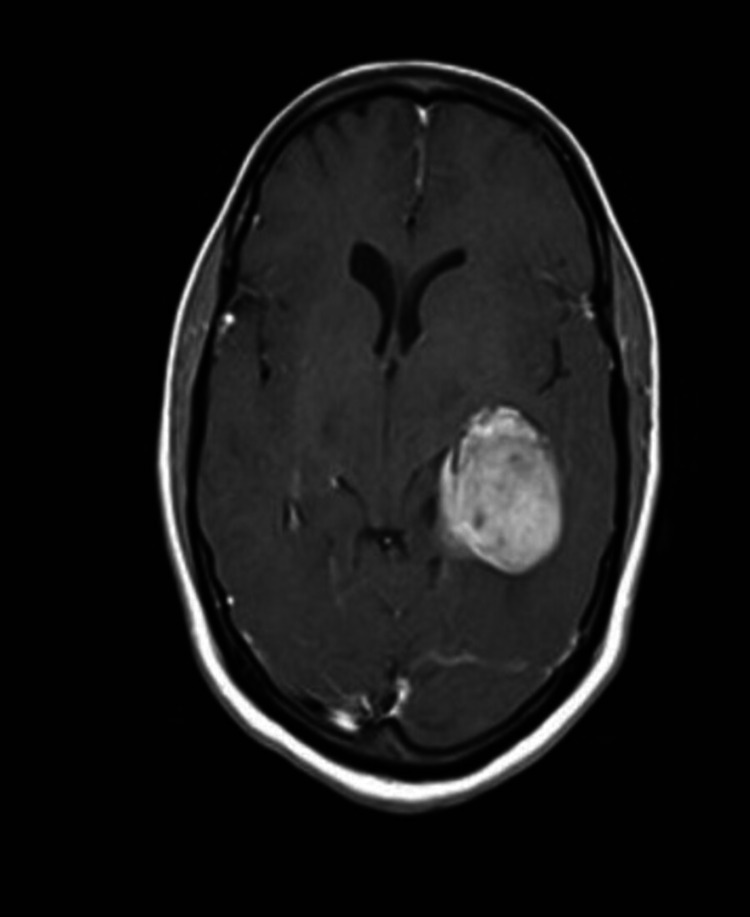
Preoperative MRI brain contrast - A

**Figure 2:**
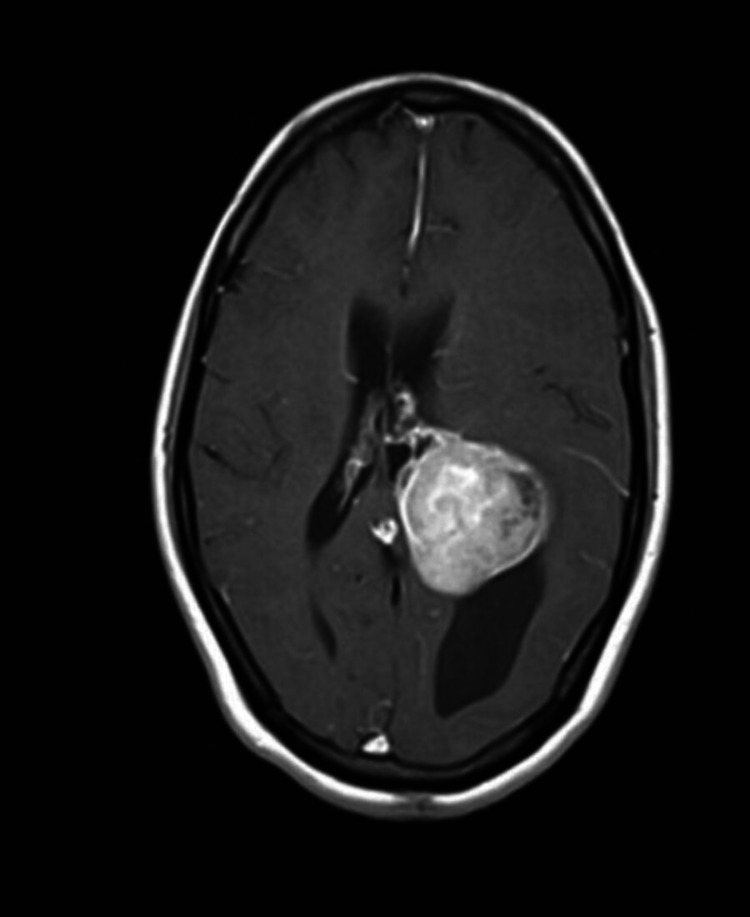
Preoperative MRI brain contrast - B

A 47-year-old female presented with a history of headache for four months and four episodes of generalized seizures. There were no visual disturbances, limb weakness, sensory deficits, behavioral changes, speech disturbances, or cranial nerve deficits. MRI brain showed large well defined lobulated T1 isointense, T2/FLAIR heterogeneously hyperintense intraventricular mass lesion arising from the atrium of left lateral ventricle, and intense heterogeneous enhancement on contrast with moderate peri-lesion edema in left parieto temporal white matter with mass effect (Figures 1, 2). CT cerebral angiogram showed arterial feeders from the left posterior cerebral artery and venous drainage into a left internal cerebral vein.

**Figure 3:**
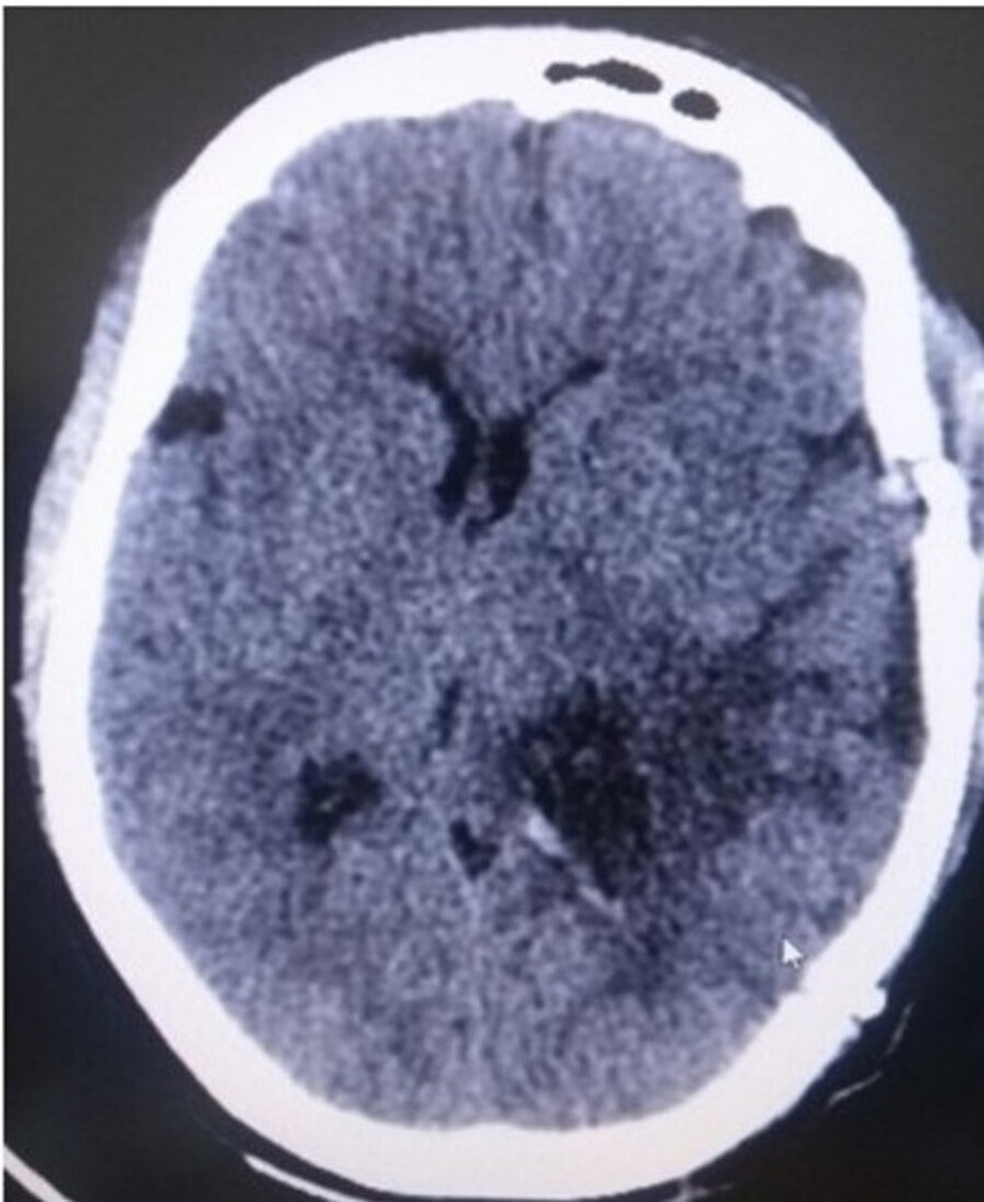
Postoperative CT scan brain - A

**Figure 4:**
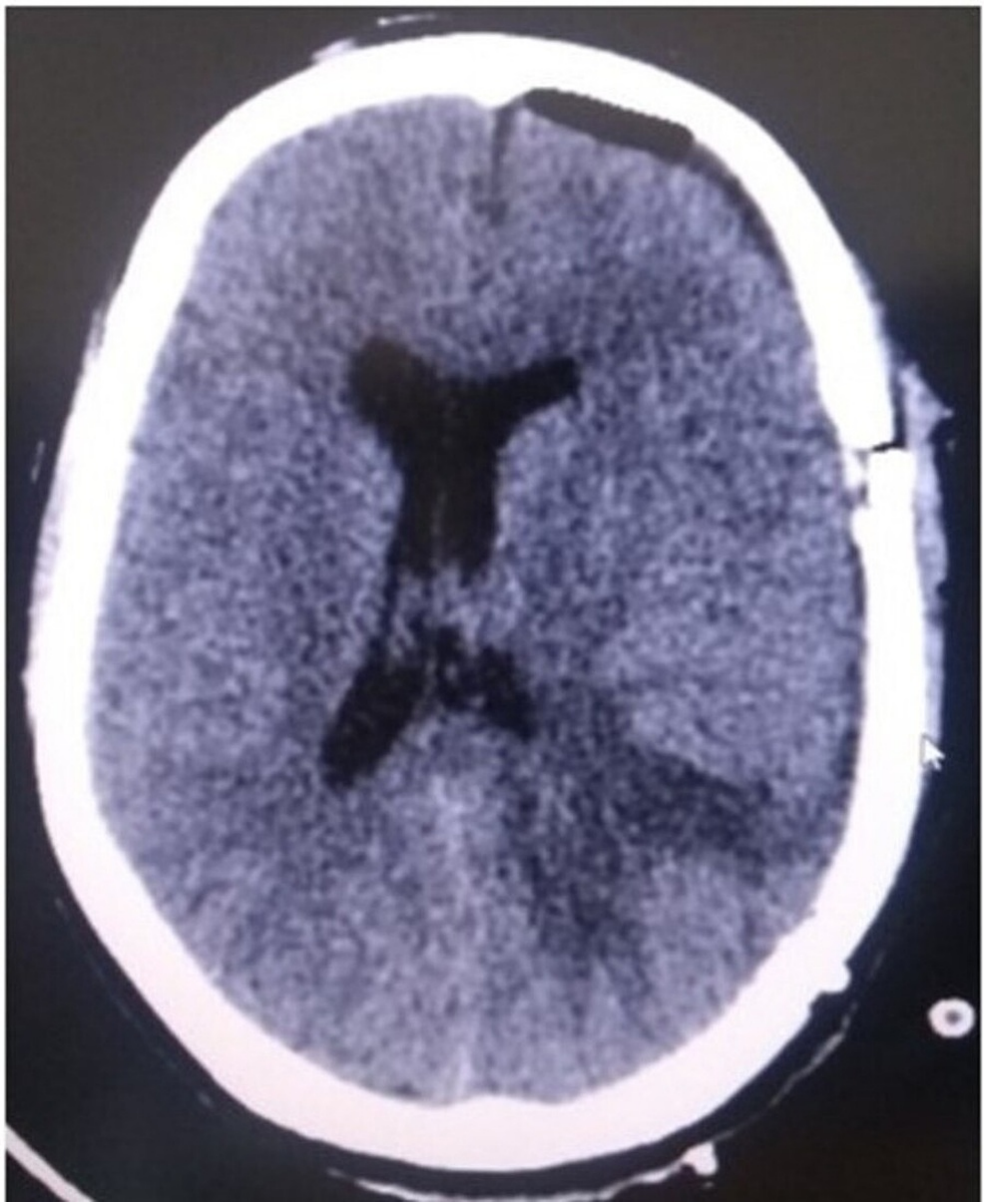
Postoperative CT scan brain - B

Left temporal craniotomy was done and a modified middle temporal gyrus approach (middle one-third) was used as it was considered the safest with minimal risk of manipulation to the neighboring structures. With this total excision of the lesion was successfully achieved. Postoperative CT scan was done to rule out any injuries to the neighboring structures, hematomas or any other complications acquired during the surgery (Figures 3, 4). Histopathological examination was diagnostic of meningioma, transitional type, WHO Grade I.

The patient postoperatively developed motor aphasia. On evaluation, aphasia was pure motor (Broca) type which is unusual considering the location of the tumor and approach used. The aphasia was transient with complete resolution over three weeks.

## Discussion

Aphasia is a language impairment, affecting speech components. There are six components of speech, i.e., fluency, comprehension, naming, repetition, reading, and writing. Edwin Smith Papyrus reported the first case of aphasia in a person with traumatic injury to the temporal lobe. Its spectrum ranges from an inability to retrieve the names of objects, or put words together into sentences, or read to being completely inarticulate.

In Wernicke's aphasia patients have intense comprehension deficits with an intact motor component. Henceforth, it is also called ‘fluent’ or ‘receptive aphasia’. Reading, writing, repetition, naming are impaired and they speak non-existent or irrelevant words without knowledge.

Conduction aphasia is an uncommon type with a characteristic debt in repetition with coherent (yet paraphasic) speech production.

Transcortical aphasias include motor, sensory and mixed transcortical aphasia. Their characteristic feature is preserved repetition. People with transcortical motor aphasia typically have intact comprehension and impaired speech production. People with sensory and mixed transcortical aphasia have poor comprehension and are unaware of their errors.

Expressive aphasia, also known as Broca's aphasia, is characterized by a loss of capacity to produce language (spoken, manual, or sometimes written), although comprehension generally remains intact. They manifest labored spontaneous speech. Speech generally contains important words conveying in very short phrases known as “telegraphic speech”. It is caused by acquired damage to the anterior regions of the brain, such as the left posterior inferior frontal gyrus or inferior frontal operculum, also described as Broca's area (Brodmann area 44 and 45).

The following tabulation has been attempted to simplify the understanding of commonly known aphasias for a better understanding of the case (Table 1).

**Table 1:** Types of aphasias

Types	Area	Fluency	Comprehension	Repetition	Naming	Reading	Writing
Broca’s	Posterior inferior frontal gyrus	-	+	-	-	-	+/-
Wernicke’s	Posterior superior temporal gyrus	+	-	-	-	-	-
Global	Large lesion involving both Broca’s and Wernicke’s area	-	-	-	-	-	-
Conduction	Lesion between Broca’s and Wernicke’s	+	+	-	+	+	+
Anomic	Associated with any aphasia	+	+	+	-	+	+
Transcortical motor	Anterior to Broca’s area	-	+	+	-	-	-
Transcortical sensory	Posterior to Wernicke’s area	+	-	+	-	-	-
Transcortical mixed	Preserved both anterior and posterior speech area but disconnected from rest of brain	-	-	+	-	-	-

In our case there was preserved comprehension; reading, naming, speech, and repetition were impaired. These features are symbolic of pure motor aphasia. As described above, the tumor was resected by a modified middle temporal gyrus approach. According to standard learning, the posterior temporal region is a seat of sensory aphasia/conduction aphasia/sensory type subcortical aphasia, and therefore the expected dysfunction with this approach was sensory or transcortical aphasias and pure motor aphasia was challenging to explain.

Marie in his study in 1906 showed that Broca's aphasia could infrequently develop without compromising the left inferior frontal gyrus [5]. Similarly, in our case tumor was approached through the posterior part of the middle temporal region, yet the patient had Broca's aphasia. In a recent article in 2007, Fridriksson et al. illustrated that Broca's aphasia developed without damage to the classical Broca's area [9]. This drives to a conclusion that Broca's area is not the only area whose injury may result in motor aphasia and there could be some other unexplored areas for word output. This theory could explain motor aphasia in our case.

In 2015, Fridriksson et al. in another article explained that a chronic Broca's aphasia is provoked not only due to damage to Broca's area but also to the left superior temporal gyrus [11]. There is no data to date demonstrating that lesions around the posterior aspect of the middle temporal area are associated with Broca's aphasia.

Okada and Hickok in 2006 showed that the superior temporal gyrus is involved in both speech perception and production [12]. But in our case, the lesion was approached through the middle temporal gyrus which is distant from the superior temporal gyrus. Even if retraction injuries to the superior temporal gyrus were to be considered as a reason for aphasia, there had to be a certain amount of sensory deficits. But our patient had no sensory deficits.

Our case is the first of its kind with transient motor aphasia due to intervention involving the posterior aspect of the middle temporal gyrus. Hence we intend that the middle temporal gyrus could also have a role in speech articulation and not just the usual posterior inferior frontal gyrus or recently described superior temporal area.

## Conclusions

From the above case, we can conclude that motor speech is more widely distributed than just primarily in the dominant frontal lobe. Patients can develop motor aphasia despite the lesion being distant from the classical posterior inferior frontal gyrus. The middle temporal gyrus could have a role in the motor language though there is no literature supporting this. Hence this could be an anatomically variant of Broca's aphasia.

Such rare studies help one to focus on decision making and help clinicians encountering such situations to act accordingly.
